# Three-Dimensional Finite Element Investigation into Effects of Implant Thread Design and Loading Rate on Stress Distribution in Dental Implants and Anisotropic Bone

**DOI:** 10.3390/ma14226974

**Published:** 2021-11-18

**Authors:** Dawit-Bogale Alemayehu, Yeau-Ren Jeng

**Affiliations:** 1 Department of Biomedical Engineering, National Cheng Kung University (NCKU), Tainan 70101, Taiwan; p88087063@gs.ncku.edu.tw; 2 School of Smart Semiconductor and Sustainable Manufacturing, National Cheng Kung University (NCKU), Tainan 70101, Taiwan; 3 Medical Device Innovation Center (MDIC), National Cheng Kung University (NCKU), Tainan 70101, Taiwan

**Keywords:** quasi-static load, abutment screw, dental implant, finite element method, dynamic load, mesiodistal

## Abstract

Variations in the implant thread shape and occlusal load behavior may result in significant changes in the biological and mechanical properties of dental implants and surrounding bone tissue. Most previous studies consider a single implant thread design, an isotropic bone structure, and a static occlusal load. However, the effects of different thread designs, bone material properties, and loading conditions are important concerns in clinical practice. Accordingly, the present study performs Finite Element Analysis (FEA) simulations to investigate the static, quasi-static and dynamic response of the implant and implanted bone material under various thread designs and occlusal loading directions (buccal-lingual, mesiodistal and apical). The simulations focus specifically on the von Mises stress, displacement, shear stress, compressive stress, and tensile stress within the implant and the surrounding bone. The results show that the thread design and occlusal loading rate have a significant effect on the stress distribution and deformation of the implant and bone structure during clinical applications. Overall, the results provide a useful insight into the design of enhanced dental implants for an improved load transfer efficiency and success rate.

## 1. Introduction

Dental implants have become increasingly common as a method for replacing missing teeth in recent decades [1,2,3,4]. However, while some studies have reported an implant success rate as high as 78–100% [5], other studies have indicated that single tooth replacement failures may occur for a variety of reasons [6], including implant surface, implant design, and bone quality factors [7] and early bone loss in the dental implant region [8]. In practice, the bio-structure of dental implants is of critical importance in determining the success rate of the implant procedure since it affects the bone directly and causes the stress distribution to change from constant to a variable, thereby putting both the implant and the bone at risk of biomechanical overload failure [9]. The success rate of dental implants is also crucially dependent on the efficiency of the stress transfer from the implant to the supporting bone [10,11,12], which depends in turn on many factors, including the loading condition [9], the implant thread design [2,13,14], and the bone material properties [15]. 

The Finite Element Method (FEM) provides an extremely efficient approach for analyzing biomechanical problems [16,17,18,19,20,21,22]. FEM is particularly attractive for the analysis of biomechanical processes, which are difficult (if not impossible) to examine in vivo or in vitro. As a result, it has been used extensively in the literature to evaluate the stress and deformation behavior of dental implants to improve their success rate [3,12,23,24,25,26,27,28,29,30,31,32,33,34]. Tribst et al. examined the mechanical performance of an alternate design for producing an implant-supported full-arch dental prosthesis (IFDP) with limited occlusal height using a finite element method (FEM) [35]. Additionally, a linear static structural finite element method (FEM) simulation was used to evaluate entire tooth models (enamel, dentin, and pulp) which is constructed with an efficient and new method using micro-computed tomography (CT) data [36]. The study by Mosavar et al. was inspired by a paucity of numerical models of bone-implant interactions for diverse osseointegration and the simplification of bone as an isotropic material in prior investigations. Further, four different thread form implants were modeled and subjected to static occlusal load and varied osseointegration conditions of the bone-implant interface using finite element analysis. It is found that the cervical cortical bone region received the most stress, as did the first thread [37]. 

During their service lives, dental implants are subjected to both static and dynamic loads [38]. Static loads are exerted on the rigid maxilla from the unmoving mandible, and the intensity is not varied over time [39]. One of the many shortcomings in Finite Element analysis is the assumption of unrealistic (static) loads in dental implants [40]. Most previous studies only consider the effects of static loads applied to a single point on the implant surface [24,41,42,43]. As a result, the induced stress does not usually exceed the yield strength of the dental implant material (e.g., 550 MPa for titanium, [44]) or bone (190 MPa for cortical bone, and 10 MPa for cancellous bone [44]). Therefore, in comparison to more realistic cyclic loading, static loads are associated with a less critical effect, creating less stress in the dental implant system [45]. Similarly, quasi-static loading has no significant influence on both implant and surrounding bone. Gehrke et al. [46] investigated one-piece and two-piece sintered dental implants subjected to quasi-static loading at a 30 degrees angle to the implant axis. The results showed that both implants could resist the loads produced during mastication. 

However, to mimic the actual mandible movement for FEM simulation, the masticatory occlusal load applied to the crown surface must be cyclic, repeatable, and dynamic with time [47]. Furthermore, cyclic loading increases stress, which was already at a peak in static loading conditions. Hence, increasing the possibility of implant fatigue and fracture during clinical service. Regardless of those consequences, not many studies about the effect of dynamic loading on the performance of dental implants and bone. Gotfredsen et al. [48,49,50] showed that in practical implant situations, high dynamic loads are much more destructive than static loads, and therefore have a critical effect on the implant success rate. The results showed that dynamic loading produced higher maximum stress than static loading and had a greater effect on the stress range as the elastic modulus varied. Bulaqi et al. [44] showed that for short dental implants subject to dynamic loads, a greater crown height space (CHS) contributed to screw loosening and fatigue fracture. Kayabaşi et al. [51] showed that for dental implants with a buttress thread shape, dynamic loading increased the stress within the implant by as much as 10–20%. 

The shape of the implant thread has a significant effect on both the stress distribution within the implant and the marginal bone rehabilitation [10]. Accordingly, an appropriate design of the thread pattern is essential to improve the uniformity of the stress distribution and reduce the stress intensity at the bone-implant interface (BII) [13,52]. Mosovar et al. [37] examined the effects of four different thread shapes on the stress distribution in the anisotropic bone under static loading conditions and found that a square thread resulted in improved performance. Many thread designs are available for dental implants, including square, triangular, buttress, reverse buttress, and trapezoidal. The thread design and associated parameters (e.g., face angle, thread width, thread pitch, and so on) have a critical effect on both the type of force produced at the BII (i.e., tensile, compressive, or shear) and the efficiency with which the load is transferred to the bone [37,53]. For example, triangular threads generate a higher shear force, while square-shaped threads produce a lower shear force and higher compressive force [52]. For square and buttress threads, the axial load is dissipated mainly through compressive forces [37,54,55]. However, for triangular and reverse buttress threads, the load is transferred through a combination of shear forces, compressive forces, and tensile forces [53]. Among the various thread designs, the square design reduces the maximum von Mises stress [56]. Albrektsson et al. [57] showed that the stress concentration of dental implants can be further reduced by curving the tops of the threads. Hansson and Werke [52] similarly showed that the maximum stress induced in the bone, and the ability of the implant to bear the load depends not only on the thread design (e.g., triangular, square, buttress, and so on) but also on the detailed parameters of the thread design, including the pitch, thread width, and face angle.

The bone material property also has an important effect on the stress distribution in dental implant systems. However, the accurate modeling of bone-related organs using finite element modeling (FEM) methods is challenging due to their inherent inhomogeneous and anisotropic characteristics [1,4,37,58]. As a result, almost all previous studies consider the implanted bone to be isotropic, homogeneous, and linearly elastic [3,4,25,41,59,60,61,62,63]. However, such a simplification results in significantly lower stress predictions for the peri-implant bone than those observed in practice [8]. Consequently, the usefulness of the simulation results for practical clinical purposes is greatly impaired.

This study aims to investigate the biomechanical behavior of five dental implants with varied thread designs when loaded under three different loading rates, as well as the impact on implant stress distribution and surrounding anisotropic bone. Additionally, we will investigate whether dynamic load will noticeably increase stresses and deformation in all five implant models and bone compared to static and quasi-static loading conditions.

## 2. Materials and Methods

### 2.1. Implant and Bone Models

**Figure 1:**
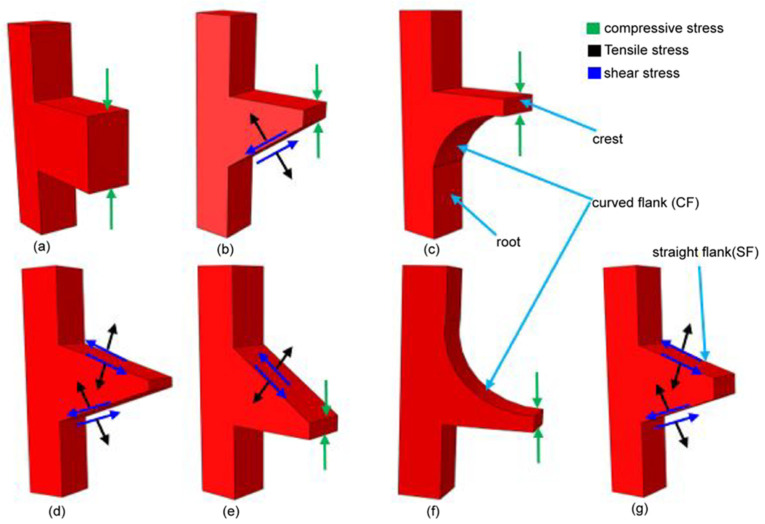
Thread design models and directions of transferred loads, (**a**) Square, (**b**) buttress (SF), (**c**) buttress (CF), (**d**) trapezoidal, (**e**) reverse buttress (SF), (**f**) reverse buttress (CF), and (**g**) triangular.

**Figure 2:**
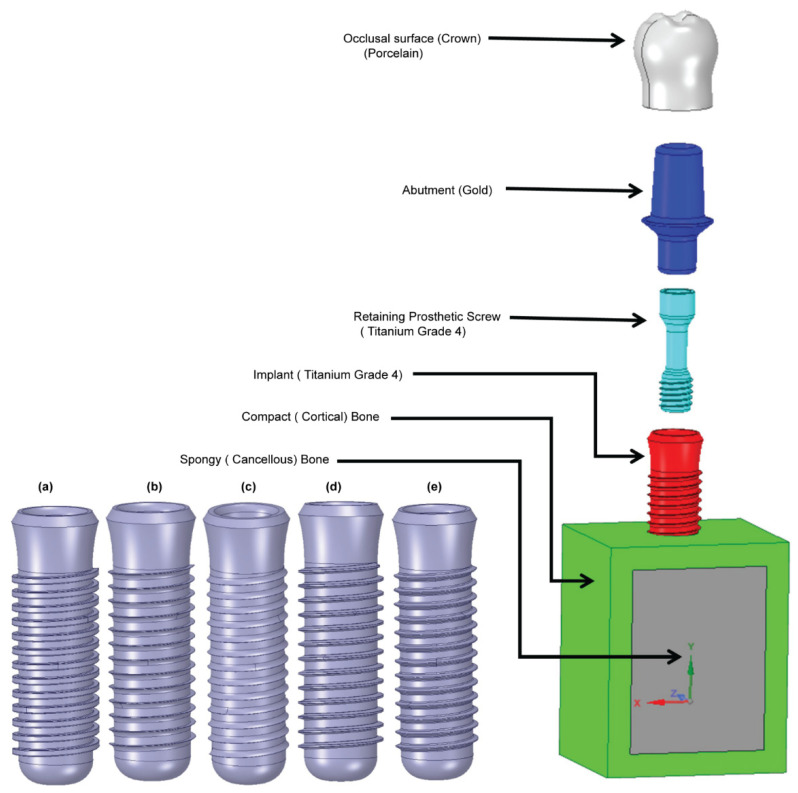
Shows the 3D models of implants and bone: (**a**) square, (**b**) buttress (CF), (**c**) reverse buttress(CF), (**d**) trapezoidal, and (**e**) triangular.

The present study performs a comprehensive FEM investigation into the effects of five different dental implant thread designs (square, buttress, reverse buttress, trapezoidal, and triangular) on the stress distribution induced within the implant and surrounding bone under three different loading rates, namely static, quasi-static and dynamic. The simulations consider the implant to consist of four components, i.e., the crown, abutment, screw, and implant. For both buttress designs, two different flank profiles were implemented, namely straight flank (SF) and curved flank (CF). Thus, as shown in Figure 1, a total of seven three-dimensional (3D) implant models were constructed, (note that for clarity of presentation, only the first thread in each model is shown). Moreover, the implanted bone is modeled as an anisotropic structure consisting of a spongy bone interior (cancellous bone) and a compact bone exterior (cortical bone). The 3D model for this investigation was made from a rectangular slice of compact and spongy bone obtained from a human jawbone (Mandible). For each model, the implant length, diameter, and pitch were specified as 14 mm, 4.1 mm and 0.8 mm, respectively, following the design specification of the commercial Straumann^®^ Standard Plus (SP) dental implant (Straumann, Basel, Switzerland). As shown in Figure 2, the prosthetic was assumed to consist of four components, namely the crown, abutment, screw, and implant. Finally, the simulations consider the implant to be jointly loaded by three external forces acting in the mesiodistal, buccal-lingual, and apical directions, respectively. The simulations focus specifically on the von Mises stress, displacement magnitude, shear stress, compressive stress, and tensile stress under each of the considered thread designs and loading conditions. The results are expected to be of significant benefit in the design of dental implants with an improved success rate under realistic clinical conditions. 

### 2.2. Material Properties and Mesh

**Figure 3:**
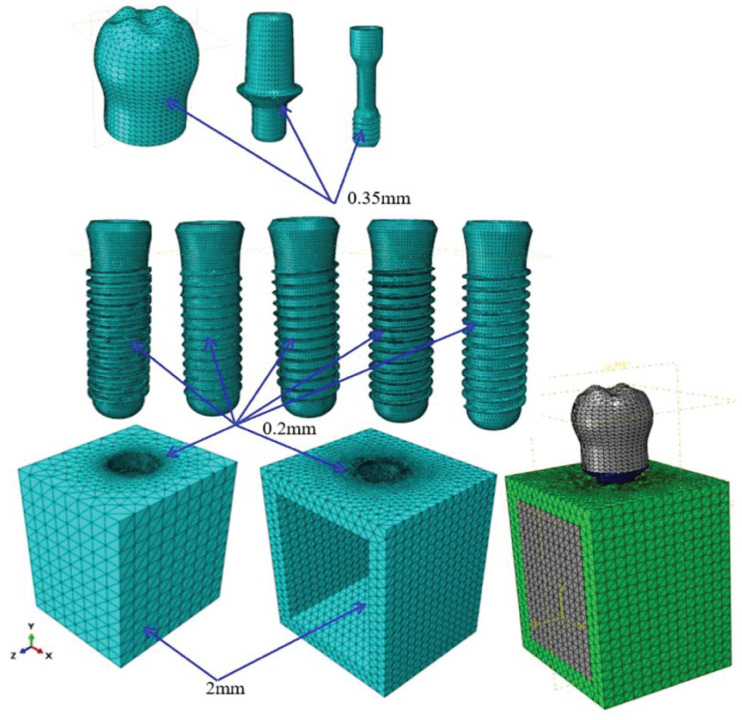
Computational meshes for implant and bone models.

The static and quasi-static responses of the dental implants were investigated using ABAQUS Standard simulations, while the dynamic response was investigated using ABAQUS Explicit. The implants and bone models were discretized using a free meshing technique with C3D10M 10-node modified quadratic tetrahedron elements. As shown in Figure 3 the models were meshed using a 0.2-mm element size in regions of the computational domain with a high-stress concentration, and 0.35-mm or 2-mm size elements elsewhere. Table 1 presents the mesh statistics of the five basic implant models. The mechanical properties of the implant and bone materials are shown in Table 2. It is noted that the mechanical properties of the crown, abutment, screw, and implant component are all assumed to be elastic, isotropic, and homogenous.

### 2.3. Load and Boundary Conditions

**Figure 4:**
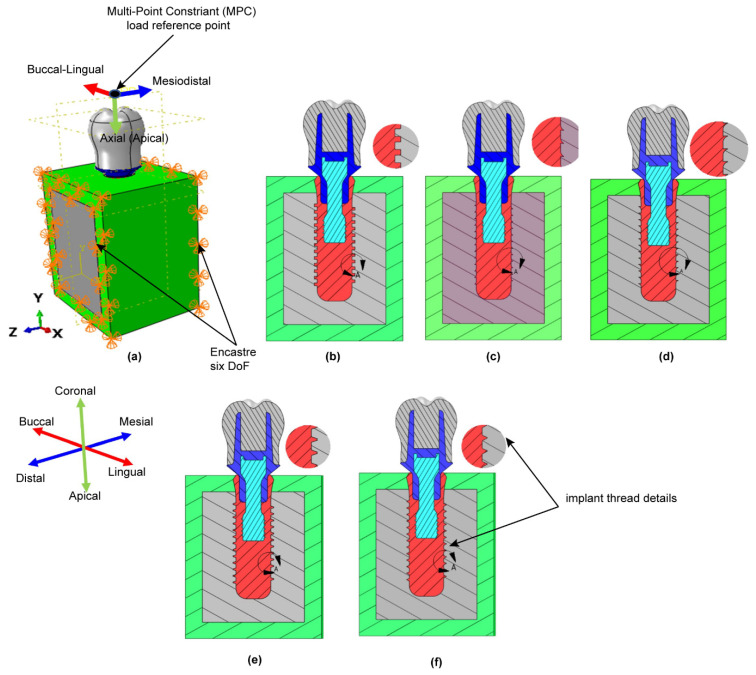
(**a**) Loading and boundary conditions. Sectional views of: (**b**) square thread, (**c**) buttress (CF) thread, (**d**) reverse (CF) buttress thread, (**e**) trapezoidal thread, and (**f**) triangular thread.

As shown in Figure 4a, the occlusal surface of the dental implant was subjected to a combined loading condition in the mesiodistal, buccal-lingual and apical directions. In performing the simulations, the magnitudes of the loads in the three directions were set as 23.4 N, 17.1 N, and 114.6 N, respectively, and were applied to a dummy reference point located at a distance of 3 mm from the occlusal surface using a multi-point constraint (MPC) technique. The resulting equivalent load was equal to 118.2 N at an angle of 75.8 degrees to the occlusal plane [44,51,64,65]. As described in the previous section, the simulations considered three different loading rates, namely static, quasi-static and dynamic. In the latter case, the external load was applied for 0.5 seconds to replicate the natural mastication cycle, which typically has a frequency of 2 Hz [44,66]. Figure 4b–f present cross-sectional views of the five basic thread designs considered in the present study. In performing the simulations, a zero-displacement boundary condition was initially applied at the interface between the implant and mandible bone in the X-, Y, and Z-directions. Moreover, to mimic the actual clinical situation at the interface between the implant and bone, frictional contact interfaces were implemented with values of 0.65 between the implant and cortical bone, and 0.77 between the implant and cancellous bone [67]. And the implants were assumed to be subjected to a combined load acting jointly in the mesiodistal, buccal-lingual, and apical directions, respectively. Moreover, the load was applied under three different rates, namely static, quasi-static and dynamic. The resulting shear stress, tensile stress, and compressive within the implant were analyzed employing finite element simulations performed in ABAQUS software (Dassault Systèmes Simulia Corporation, Providence, RI).

## 3. Results

### 3.1. Mesh Sensitivity Analysis

**Figure 5:**
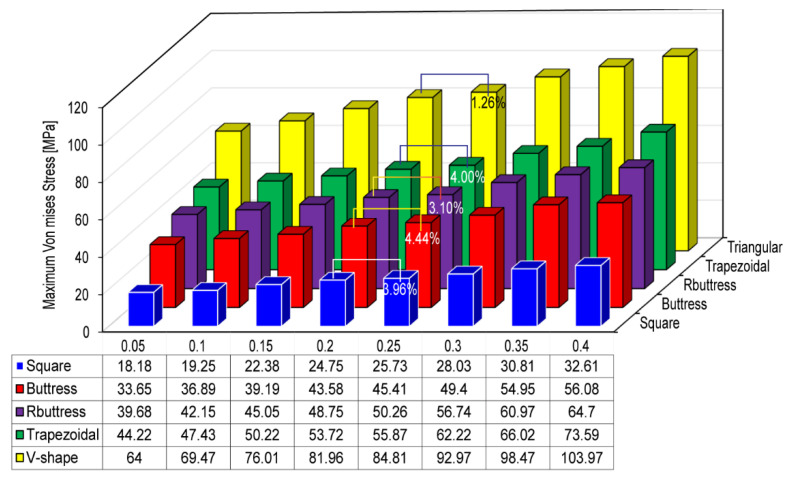
Maximum von Mises stress for different implant models and element sizes.

In performing FEA simulations, convergence tests are required to achieve an adequate tradeoff between the computational cost of the simulation process and the accuracy of the numerical solutions. Accordingly, the five implant models were implemented with eight different element sizes in the range of 0.05~0.4 mm. For each mesh size, the maximum von Mises stress was computed in the compact bone region. The simulation results were then inspected to determine the mesh size at which the change in the maximum von Mises stress was limited to less than 5%. And the convergence test was performed under a dynamic loading rate. The corresponding results are presented in Figure 5. A detailed inspection shows that the maximum von Mises stress increases by just 3.96%, 4.44%, 3.10%, 4.00%, and 1.26% as the element size increases from 0.2 to 0.25 mm in the square, buttress, reverse buttress, trapezoidal, and triangular models, respectively. Accordingly, an element size of 0.2 mm was adopted as the optimal seed size for the meshing process (see Figure 3).

### 3.2. Cyclic Load vs. Time

**Figure 6:**
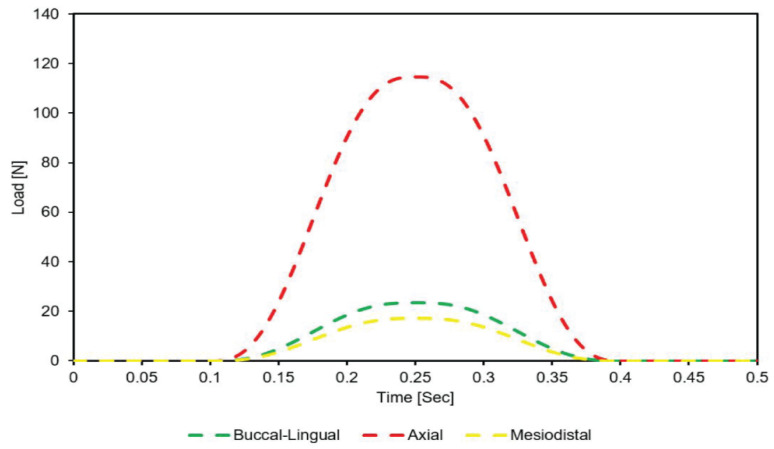
Dynamic loading of crown occlusal surface in buccal-lingual, axial, and mesiodistal directions during mastication cycle (2 Hz).

Figure 6 shows the variation of the load applied to the occlusal surface of the crown in the mesiodistal, buccal-lingual, and apical directions throughout the mastication cycle (0.5 s).

### 3.3. Maximum Von Mises Stress

**Figure 7:**
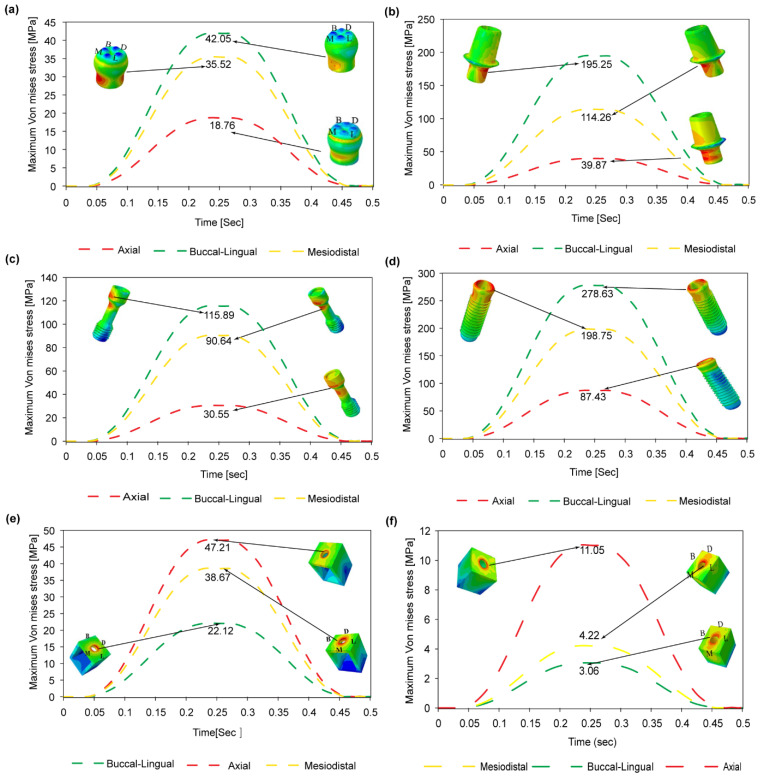
Shows the maximum von mises stress due to dynamic loading in Buttress (CF) implant in case of (**a**) Crown, (**b**) abutment, (**c**) Screw, (**d**) implant, (**e**) Compact bone, and (**f**) Spongy bone.

Figure 7 shows the corresponding variation in the maximum von Mises stress in the crown, abutment, abutment screw, Buttress thread implant, cortical bone, and spongy bone regions of the model, respectively. It is seen that for all of the implant components, the maximum von Mises stress is produced under buccal-lingual loading, followed by mesiodistal and axial loading, respectively. Moreover, the maximum von Mises stress is induced in the implant component of the prosthetic (Figure 7d). The maximum stress has a value of 278.63 MPa and is thus around 50.66% of the implant material (titanium) yield strength (see Table 1). By contrast, the minimum von Mises stress is produced in the crown region of the implant (Figure 7a). Observing the results presented in Figure 7e for the cortical bone region of the model, it is seen that the maximum von Mises stress is produced under axial loading, followed by mesiodistal and buccal-lingual loading, respectively. By contrast, for the spongy bone region of the model, the maximum stress is produced under axial loading, followed by buccal-lingual and mesiodistal loading (Figure 7f). Comparing the two figures, the maximum von Mises stress in the cortical bone region (47.21 MPa, 22.31% of the yield strength, see Table 1) is higher than that in the spongy bone region (11.05 MPa, 110% of the yield strength). Moreover, the maximum von Mises stress is distributed relatively uniformly between the three loading conditions. However, the magnitude of the maximum von Mises stress is significantly reduced and less evenly distributed in the spongy bone region (11.05 MPa under axial loading and 3.06 MPa under mesiodistal loading). 

Table 3 contains results from ONE WAY ANOVA statistical analysis comparing the effect of the three loading rates on stress in all implant models. The result showed, the *p*-value is greater than 5% and way far below the F critical value. Also, Table 4 shows the maximum von Mises stress in the crown, abutment, screw, implant, cortical bone, and spongy bone regions of the five models under the static, quasi-static and dynamic loading conditions. It is evident that for each region of the model, the maximum von Mises stress increases significantly under the application of a dynamic load. The increases are 32%, 38.19%, 45.10%, 51.20%, and 58.21% for square, buttress, reverse buttress, trapezoidal, and triangular thread implant, respectively. And Square, buttress, reverse buttress, trapezoidal, and triangular thread implants have a 32%, 38.17%, 45.08%, 51.19%, and 58.19% stress increase in cortical bone, respectively. Moreover, for each model, the maximum von Mises stress occurs in the implant region of the prosthetic. 

**Figure 8:**
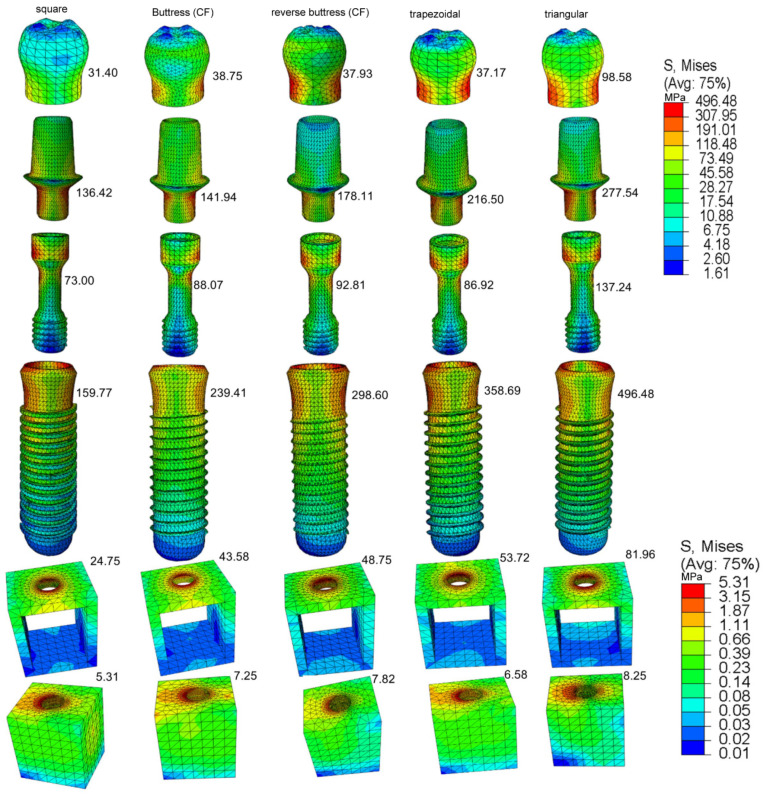
The maximum von Mises stress in the prosthetic components, all implant design, and anisotropic bone under dynamic load.

**Figure 9:**
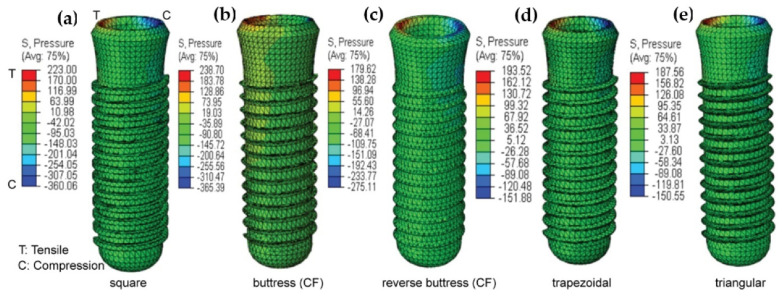
Shows the maximum pressure in (**a**) Square, (**b**) Buttress, (**c**) reverse buttress, (**d**) trapezoidal, and (**e**) triangular thread shape under cyclic loading.

Figure 8 shows the von Mises stress distributions in the crown, abutment, retaining screw, implant, and supporting bone regions of the five models under dynamic loading conditions. As shown in Figure 9c, under dynamic loading conditions, the maximum von Mises stress occurs in the implant component of the prosthetic, while the lowest von Mises’s stress occurs in the spongy bone. The maximum von Mises stress has a value of 496.48 MPa and occurs in the triangular thread model, while the lowest von Mises stress has a value of 159.77 MPa and occurs in the square thread model. Regarding the bone regions of the model, the von Mises stress has maximum values of 81.96 MPa and 24.75 MPa in the cortical bone regions of the triangular and square thread models, respectively, and minimum values of 8.25 MPa and 5.31 MPa.

Table 5 compares the maximum von Mises stresses induced in the buttress and reverse buttress models for two different flank designs (curved and straight) under each of the three loading conditions. It is seen that for each model, the application of a curved flank design lowers the maximum von Mises stress in all the prosthetic and bone regions of the model.

The reduction in the maximum von Mises stress is particularly apparent under the dynamic loading condition and in the crown region of the prosthetic, for which the reduction is equal to almost 50%. The addition of curved flank (CF) to the buttress and reverse buttress thread profiles will greatly increase compressive stress while significantly lowering tensile stress. After this result is established, only buttress and reverse buttress with CF profile are examined in this study.

### 3.4. Maximum Pressure

Figure 9 shows the maximum pressure (tensile stress and compressive stress) in the square, buttress, reverse buttress, trapezoidal, and triangular thread implant models under dynamic loading conditions. For each of the models, the maximum pressure occurs in the implant neck region, where the implant and cortical bone make contact. The maximum compressive stress is produced in the square and buttress (CF) thread implants, with magnitudes of 360.06 MPa and 365.39 MPa respectively. The addition of a curved flank to a buttress implant increased compressive stress when compared to a square implant. By contrast, the trapezoidal and Triangular thread designs result in relatively lower maximum compressive stresses of 151.88 MPa and 150.55 MPa, respectively. 

### 3.5. Maximum and Minimum Principal Stress

**Figure 10:**
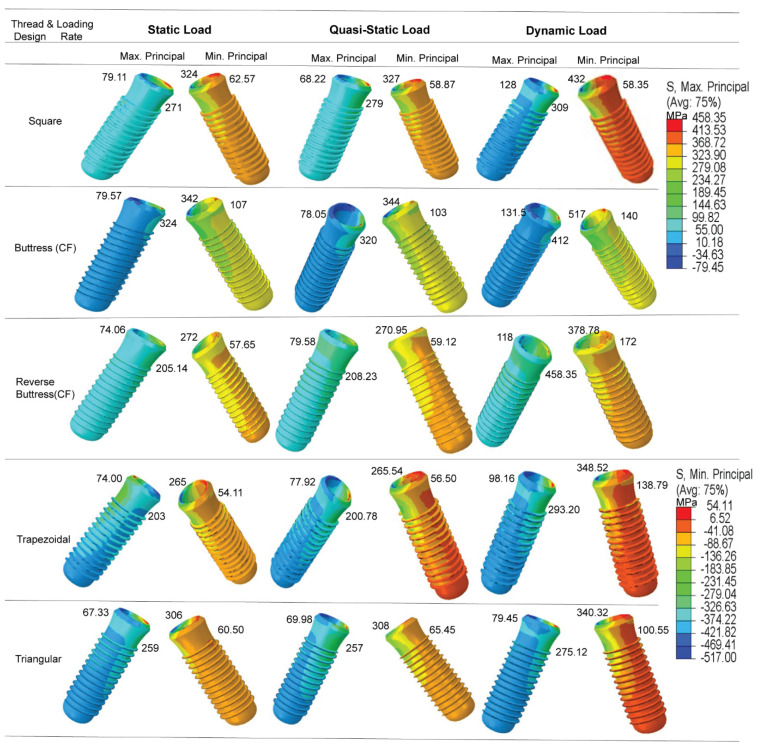
Maximum and minimum principal stresses in implant under dynamic load.

The results confirm that the dynamic loading condition yields a significant increase in the magnitude of the principal stress in all five implant models (Figure 10). The percentage change in minimum and maximum compressive principal stresses due to dynamic loading for buttress thread implant is 51.17% and 65.26% and for reverse buttress implant is 39.26% and 59.33%, respectively. However, the static and quasi-static loading conditions show no significant difference in effect on the stress response of the implants. The highest maximum principal (tensile) stress occurs in the reverse buttress (CF) model and has a value of 458.35 MPa. By contrast, the highest minimum principal (compressive) stress occurs in the buttress thread (CF) model and has a value of 517 MPa. For both stresses (tensile and compressive), the maximum stress occurs in the tip bevel region of the implant where it contacts the crestal part of the cortical bone.

### 3.6. Maximum Displacements

**Figure 11:**
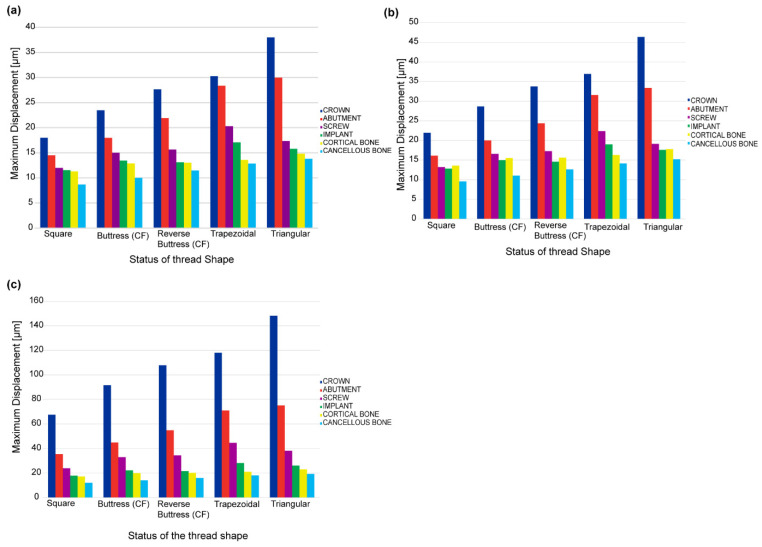
Maximum displacements of prosthetic and bone regions of different thread models under (**a**) static loading, (**b**) quasi-static loading, and (**c**) dynamic loading.

Figure 11a–c show the maximum displacements of the crown, abutment, screw, implant, cortical bone, and spongy bone regions of the five models under static, quasi-static, and dynamic loading conditions, respectively. For all the thread models, and each of the three loading conditions, the maximum displacement occurs in the crown region of the prosthetic, while the minimum displacement occurs in the spongy bone. Furthermore, for each loading condition, the square thread design results in the lowest displacement, while the trapezoidal and triangular designs yield the highest displacement. Comparing the three figures, it is seen that the quasi-static and static loading conditions yield a similar maximum displacement of the prosthetic and bone regions of the model. However, the dynamic loading condition yields a significant increase in the maximum displacement of all regions of the model for all five thread designs. In square thread implants, the percentage change in maximum displacement because of applying dynamic load is 54%, and 65% for the rest of the dental implant thread type. Maximum displacement changes attributed to dynamic load in cortical bone were 52.03% in square thread implants, 41.76% in triangular thread implants, and 55% in the other three implants. 

### 3.7. Maximum Shear Stress

Table 6 shows the maximum shear stress in the crown, abutment, screw, implant, cortical bone, and spongy bone regions of the five basic thread models for each of the three planes (XY, XZ, and YZ) and loading rate conditions (static, quasi-static, and dynamic). For each model, the maximum shear stress increases significantly on all planes under the application of a dynamic load (40–99%). The maximum shear stresses in the three planes (XY, XZ, and YZ) for the square thread implant in static and dynamic loading were (27.75 MPa, 19.69 MPa, and 17.13 MPa) and (41.66 MPa, 29.30 MPa, and 25.51 MPa), respectively. The percentage change in maximum shear stress in three planes (XY, XZ, and YZ) of the dynamic loading rate is shown here (50.13%, 48.81%, and 48.92%). Similarly, in triangular thread implant, the percentage change in the maximum shear stress in the three planes (XY, XZ, and YZ) of the static and dynamic loading rate is (95.56%, 85.67%, and 99.14%). The *p*-value for the one-way ANOVA statistical analysis in Table 7 is greater than 5%. As a result, there is no discernible difference in the stress responses of the various components within each model under static and quasi-static loads. Furthermore, based on the results of the ANOVA analysis (Table 8), the square thread and buttress thread models produce lower shear stress values than the reverse buttress, trapezoidal, and triangular models. Moreover, for all thread models and loading directions, the shear stress in the cortical bone is greater than that in the spongy bone. And the effect of dynamic load on shear stress was significant but varied in every three planes (XY, XZ, and YZ planes).

## 4. Discussion

Based on the ONE WAY ANOVA statistical analysis result *p*-value was greater than 5%, indicating that the result is statistically non-significant (Table 3). In other words, there is a remarkable increase in stress magnitude in the case of dynamic loading compared to that of quasi-static and static loading conditions in all five implant models. Also from the FEA result and based on the approach used in this study, the dynamic load applied to the crown surface increased the stresses by 30–60% compared to unrealistic (static) loading (Table 4, Table 5 and Table 6) which is highly significant. Dynamic mechanical loading can damage the surface morphology and chemistry of dental implants [68] and can result in the penetration of micro-organisms down to the threaded region of the fixture-abutment interface [69]. Furthermore, cyclic loading can increase the stress induced in the implant by around 10–20% compared to that observed under static loading [51]. Previous studies have shown that the stress induced in the implant and surrounding bone region depends not only on the nature of the applied load (i.e., static or dynamic) but also on the design of the implant thread [70,71]. 

It has been shown that for all five thread design models, the von Mises stress, shear stress, compressive stress, and displacement all increase significantly under a dynamic load compared to a static or quasi-static load. However, little difference has been observed in the stress response of the implant under static and quasi-static loads, respectively. In general, the results have shown that the maximum stress is concentrated in the contact areas of the implant-bone models, e.g., at the abutment-screw, abutment-implant, and bone-implant interfaces (see Figure 7 and Figure 9). Accordingly, for all thread designs, there is a risk of micromotion, which may cause screw loosening, crestal bone loss, and implant fracture. The present results have shown that, under dynamic loading, the maximum von Mises stress in the prosthetic components is higher under buccal-lingual loading than under axial or mesiodistal loading (see Figure 7a–d). By contrast, in the cortical and spongy bone regions, the maximum von Mises stress is produced under axial compressive loading (see Figure 7e,f). In the case of trabecular bone, its yield strength threshold is exceeded under dynamic stress in the axial direction. This increase indicates that trabecular tissue yield strength is higher in compression than in tension. This could result in bone resorption and mineral redistribution to help with bone remodeling. Due to higher stress carrying in the implant, followed by cortical bone, the Von Mises stress is lower in spongy bone for all loading rates.

The results presented in Figure 9b have shown that the maximum principal stress is concentrated in the first thread of the implant in the tip bevel region where it contacts the crestal part of the cortical bone. This prediction is consistent with the findings of Sun et al. [72] that the abutment screw commonly breaks at the first thread and the implant breaks in the neck region. The simulation results obtained for the maximum shear stress (Table 6) have shown no significant differences in the stress response behaviors of the five thread models under static loads and quasi-static loads, respectively. However, for each model, the stress increases significantly under dynamic loading. When all implant models were considered, it increased by 30–60% (see Table 4 and Figure 9c). This is extremely important because it implies that such cyclic loading rates must be closely monitored, since they may cause implant and bone fatigue and fracture. Many previous studies have reported that dynamic loading can have a greater effect on the implant success rate than static loading. For example, Yagihara et al. [65] found that the attachment strength was significantly improved following the application of a dynamic load for four weeks compared to that obtained under a static load after four or eight weeks. Likewise, Duyck et al. [73] reported that under dynamic stress situations, peri-implant bone resorption performance in rabbit tibias was significantly better than it was under static load scenarios. 

Furthermore, previous studies have generally considered only a static masticatory load, [37,74,75,76,77,78,79,80,81,82]. However, in addition to static load, the current study considered quasi-static and dynamic loading rates. As a result, this research yields better results and a more reliable conclusion. This also reveals that dynamic loading considerably increases stress in all five models and cortical bone, potentially leading to bone fatigue and fracture. The results obtained in the present study have shown that the application of dynamic loading can increase the stress of dental implants and cortical bone by as much as 30–60% compared to static loading (see Table 4 and Figure 9c). This percentage increment is obtained when considering all the five implant models. And most importantly, including buttress and reverse buttress implants with CF thread profiles. Kayabaşi et al. [51] considered only a single dental thread design (reverse buttress), whereas the present study has considered five different models. The present results have shown that while dynamic loading yields an effective increase in the compressive stress in the implant and bone region and is therefore beneficial in improving the implant success rate, the thread design should be carefully chosen to avoid failure in the first thread region of the implant.

The FEA results obtained in the present study have shown that, for all the loading rates and thread designs, the maximum von Mises stress is concentrated in the implant region of the prosthetic (see Table 4 and Figure 9c). As a result, the minimum stress is transferred to the bone, and the uniformity of the stress distribution within the bone is improved. The present results are thus in good general agreement with those of previous studies [37,51,83,84,85,86]. However, most previous studies did not consider the crown and abutment screw components of the prosthetic [37,83,85,86], and hence, the clinical application of the FEA findings is somewhat limited. Adding the mechanical retaining screw and occlusal surface enhanced the reliability of the result from FEM and mimicking the clinical situation during mastication load is applied by the patient.

The present results have shown that the implant thread design affects both the magnitude and the distribution of the stress induced in the cortical bone under all three loading rates (see Table 4 and Figure 9c). They are thus in good agreement with the findings of Hansson and Werke [52]. In particular, the results have shown that for the buttress and reverse buttress thread models, the application of curved flanks reduces the maximum von Mises stress by up to 50% compared to straight flanks (Table 5), curved flank provides more bone contact area by decreasing the shear stress and tensile stress. Hence, more bone contact area has been proven to offer increased initial stability and stress resistance [87]. Among the various thread designs considered in the present study, the square thread design results in the lowest von Mises stress, shear stress, and displacement, and the highest compressive stress (see Figure 8 and Figure 9b, Table 4 and Table 6). In general, the masticatory load acting on the crown occlusal surface results in three different stresses, namely tensile, compressive, and shear. Tensile stresses tend to pull objects apart, and if it becomes more than the yield strength, it may cause failure in implant and bone loss, while shear forces promote sliding. By contrast, compressive stresses maintain the integrity of the bone-implant interface. Furthermore, cortical bone is the strongest in compression and the weakest in shear [88]. Thus, the present results suggest that the square thread design, which enhances the compressive stress, is the most suitable for dental implant applications. However, the buttress and reverse buttress designs with curved flanks also reduce the tensile and shear stress, while also promoting compressive stress (see Figure 9 and Table 5). Consequently, the buttress thread designs with curved flanks also represent a favorable design for dental implants. Furthermore, for each design, the maximum displacement, von Mises stress, and shear stress are significantly lower in the spongy bone region than in the cortical bone region (see Figure 8 and Figure 9, and Table 6).

The other factor that directly affects the biomechanical behavior of dental implants is a bone material property. Unlike this study, most previous studies considered bone as an isotropic model. Martins et al. designed 2D computational axisymmetric models of isotropic bone implants and used varied thread profile shapes to anticipate the effect of material stiffness in dental implants [16]. However, human bone is appearing to be highly anisotropic and is directional dependent. Consequently, for the case of isotropic bone, the resulting stress and strain from the FEM might not represent the actual stress during clinical service. Anisotropy, according to O’Mahony et al. [89], would increase the already high levels of stress and strain in the cortical crest by 20 to 30% in the isotropic scenario. And further reported, anisotropy increased what were previously minimal levels of interface tensile and radial-hoop shear stresses in cancellous bone by three to four times along the lingual side, exceeding bond strength [89]. And As per Xi et al., percentage increases in maximum stresses of cortical bone surrounding implant in the orthotropic model were up to 13.2 percent higher than those in the functionally isotropic model, but those on the cancellous bone surrounding implant were up to 226.7 percent higher [90]. The findings of this study are consistent with previous research results. 

Although FEM is a powerful and efficient way to solve biomechanical problems, there are limitations during the analysis of this study. Dynamic analysis in this study is computationally intensive both in terms of calculation time and computer memory usage. It is a heavy task for the testing of the convergence for different element sizes against maximum stress. Quad finite element type yields excellent FEM outcome with less numerical error. But our study has employed modified quadratic tetrahedral finite element type with 10-nodes (C3D10M) due to the complexity of the implant geometry and 3D model, limited computer RAM, and convergence time.

## 5. Conclusions

In this study, the influence of different loading rates and thread design on stress distribution of dental implants inserted in anisotropic bone was studied using the FEM technique. Overall, the simulation results support the following main conclusions:

■ The application of dynamic masticatory loading to the occlusal surface of the prosthetic increases the stress within the implant and surrounding bone significantly.
■ The outcomes of the simulation revealed that while static loading analyses provide a convenient and low-complexity approach for performing the preliminary design of dental implants, dynamic loading analyses are required to properly understand the performance implications of the proposed design in clinical situations.
■ No significant difference exists between the stress response behavior of the different thread design models under static loading and quasi-static loading conditions, respectively.
■ The adoption of curved flanks for buttress and reverse buttress threaded implants resulted in a large improvement in compressive stress and a considerable reduction in maximum stress.
■ Square thread implants had more favorable stress and strain distribution, which may improve the process of bone remodeling.
■ We also infer that the anisotropic bone behavior has an unavoidable consequence on the stresses and strain distribution, which could improve the FEM result’s robustness. And it can be used to replace complex bone models, which are often computationally expensive.

## Figures and Tables

**Table 1:** Mesh statistics of different implant and bone models.

	Crown		Abutment		Screw		Implant		Cortical Bone		Trabecular Bone		Total	
Thread Shape	No.Element	No.Node	No.Element	No.Node	No.Element	No.Node	No.Element	No.Node	No.Element	No.Node	No.Element	No.Node	No.Element	No.Node
Reverse Buttress (CF)	28,366	6087	21,417	5081	16,901	3724	168,760	38,512	40,743	8972	306,173	57,166	582,360	119,542
Buttress (CF)	36,970	7809	20,725	4969	17,022	3748	161,164	37,266	41,045	9020	334,731	64,236	611,657	127,048
Square	26,370	5698	21,115	5033	16,894	3729	235,374	47,451	49,741	10,912	343,846	68,114	693,340	140,937
Triangular	28,060	6023	21,455	5097	16,876	3729	235,860	50,594	40,217	8873	373,046	70,342	715,514	144,658
Trapezoidal	27,979	5990	20,991	5014	16,937	3736	168,399	35,006	49,974	10,946	380,451	72,147	664,731	132,839

**Table 2:** Physical properties of materials for the FEA [39,59].

Materials	Young’s Modulus E (MPa)		Poisson’s Ratio ν		Density (g/cm^3^)	Strength (MPa)
Cortical bone	E_x_	12,600	ν_xy_	0.3	3	190
	E_y_	12,600	ν_yz_	0.253		
	E_z_	19,400	ν_xz_	0.253		
			ν_yx_	0.3		
			ν_zy_	0.39		
			ν_zx_	0.39		
Cancellous bone	E_x_	1148	ν_xy_	0.055	3	10
	E_y_	210	ν_yz_	0.01		
	E_z_	1148	ν_xz_	0.322		
			ν_yx_	0.01		
			ν_zy_	0.055		
			ν_zx_	0.322		
Gold abutment *	136,000		0.37		17.5	765
Porcelain	68,900		0.28		2.44	145
Titanium grade 4 *	110,000		0.34		4.5	550

* The vectors of x, y and z are mean the buccolingual, infero-superior, and mesiodistal direction, respectively. And Implant & screw = Titanium grade 4, Abutment = Gold, Crown = Porcelain.

**Table 3:** Show the result of testing the null hypothesis using ONE WAY ANOVA statistical analysis.

Anova: Single Factor						
Summary						
Groups	Count	Sum	Average	Variance		
Static	30	2467.66	82.25533333	6338.674991		
Quasi-Static	30	2593.42	86.44733333	6867.053779		
Dynamic	30	3513.29	117.1096667	14,067.48338		
ANOVA						
Source of Variation	SS	df	MS	F	*p*-value	F_crit_
Between Groups	21,725.76102	2	10,862.88051	1.194895612	0.307654	3.101296
Within Groups	790,923.1522	87	9091.070715			
Total	812,648.9132	89				

**Table 4:** Maximum von Mises stress in a different region of each model under static, quasi-static, and dynamic loads.

Maximum Von Mises Stress (MPa)				
Thread Type	Components	Static	Quasi-Static	Dynamic
Square	CROWN	23.79	24.86	31.40
	ABUTMENT	103.35	108.00	136.42
	SCREW	55.30	57.79	73.00
	IMPLANT	121.04	126.49	159.77
	CORTICAL BONE	18.75	19.60	24.75
	SPONGY BONE	4.02	4.21	5.31
Buttress (CF)	CROWN	24.29	25.56	38.75
	ABUTMENT	110.03	115.81	141.94
	SCREW	68.27	71.85	88.07
	IMPLANT	173.24	182.33	239.41
	CORTICAL BONE	31.54	33.19	43.58
	SPONGY BONE	2.25	6.23	7.25
Reverse buttress (CF)	CROWN	27.45	28.80	37.93
	ABUTMENT	128.88	135.22	178.11
	SCREW	67.16	70.46	92.81
	IMPLANT	205.79	214.42	298.60
	CORTICAL BONE	33.60	35.01	48.75
	SPONGY BONE	2.80	5.65	7.82
Trapezoidal	CROWN	27.50	28.99	37.17
	ABUTMENT	160.13	168.86	216.50
	SCREW	64.29	67.79	86.92
	IMPLANT	237.23	247.06	358.69
	CORTICAL BONE	35.53	37.00	53.72
	SPONGY BONE	4.35	4.53	6.57
Triangular	CROWN	70.82	74.00	98.58
	ABUTMENT	199.38	208.36	277.54
	SCREW	98.59	103.03	137.24
	IMPLANT	313.81	327.93	496.48
	CORTICAL BONE	51.81	54.14	81.96
	SPONGY BONE	2.67	6.25	8.25

**Table 5:** Maximum von Mises stress in buttress and reverse buttress models with curved flank (CF) and straight flank (SF) design.

Maximum Von Mises Stress (MPa)										
		SF			CF			Decrease (%)		
Thread Design	Components	Static	Quasi-Static	Dynamic	Static	Quasi-Static	Dynamic	Static	Quasi-Static	Dynamic
Buttress	crown	37.48	44.45	74.81	24.29	25.56	38.75	35.2	42.5	48.2
	abutment	147.49	168.33	246.03	110.03	115.81	141.94	25.4	31.2	42.31
	screw	97.68	96.95	142.78	68.27	71.85	88.07	30.11	25.89	38.32
	implant	245.07	234.42	402.91	173.24	182.33	239.41	29.31	28.57	40.58
	Cortical bone	41.88	42.2	68.36	31.54	33.19	43.58	24.69	21.36	36.25
	Cancellous bone	2.72	7.61	9.16	2.25	6.23	7.25	17.25	18.19	20.85
Reverse buttress	crown	37.26	43.08	23.81	27.45	28.8	37.93	26.32	33.14	49.78
	abutment	159.39	157.43	217.21	128.88	135.22	178.11	17.59	14.11	18
	screw	95.52	90.47	136.87	67.16	70.46	92.81	29.69	22.12	32.19
	implant	254.72	271.35	449.83	205.79	214.42	298.6	19.21	20.98	33.62
	Cortical bone	44.08	43.86	64.82	33.6	35.01	48.75	23.78	20.18	24.79
	Cancellous bone	3.5	7.2	10.43	2.8	5.65	7.82	19.89	21.5	25

SF = straight flank, CF = curved flan.

**Table 6:** Maximum shear stress in three different planes of basic thread models under static, quasi-static, and dynamic loading conditions.

Shear Stress [MPa] for Oblique Load-118.2N										
		Static Load			Quasi-Static Load			Dynamic Load		
Thread Type	Components	XY	XZ	YZ	XY	XZ	YZ	XY	XZ	YZ
Square	CROWN	7.15	13.15	5.91	7.51	13.61	6.04	10.69	19.32	8.67
	ABUTMENT	29.07	10.09	33.88	31.83	10.66	34.31	40.67	13.91	47.88
	SCREW	7.44	5.18	13.24	8.00	5.41	13.93	10.06	7.62	18.38
	IMPLANT	27.75	19.69	17.13	29.32	20.99	18.59	41.66	29.30	25.51
	CORTICAL BONE	4.94	8.97	3.36	5.27	9.18	3.53	7.24	13.36	5.28
	SPONGY BONE	1.01	1.08	1.06	1.01	1.14	1.10	1.61	2.16	1.99
Buttress (CF)	CROWN	4.55	3.78	7.52	4.92	4.03	7.62	6.51	5.68	11.45
	ABUTMENT	25.00	9.63	54.53	26.66	10.17	57.38	35.10	14.46	79.61
	SCREW	9.35	10.18	15.95	9.94	10.74	17.31	14.13	14.71	23.66
	IMPLANT	35.90	22.55	14.98	38.45	23.28	15.72	53.38	33.92	26.80
	CORTICAL BONE	5.71	10.73	2.40	5.73	11.22	2.49	9.69	16.32	7.21
	SPONGY BONE	0.53	0.66	0.64	0.56	0.69	0.65	0.99	1.26	2.46
Reverse Buttress (CF)	CROWN	14.26	6.72	36.69	15.31	7.21	39.45	20.68	9.94	53.29
	ABUTMENT	46.29	46.20	53.10	49.62	49.46	56.95	72.01	73.94	82.65
	SCREW	14.81	10.37	19.19	16.04	11.32	20.84	22.12	15.80	28.72
	IMPLANT	43.27	32.08	19.24	43.57	32.34	19.39	77.41	58.48	34.45
	CORTICAL BONE	6.72	12.19	2.76	7.15	13.08	2.93	12.73	24.28	8.26
	SPONGY BONE	0.63	0.90	0.58	0.69	0.99	0.63	2.26	3.39	2.12
Trapezoidal	CROWN	5.17	3.85	11.40	5.58	4.11	12.11	7.76	6.22	18.81
	ABUTMENT	26.77	15.72	55.36	28.93	17.05	59.36	41.02	24.37	84.76
	SCREW	9.83	5.26	17.11	9.89	5.36	17.25	17.66	9.47	32.56
	IMPLANT	51.59	26.71	20.61	53.89	28.07	22.03	80.55	46.23	34.09
	CORTICAL BONE	6.94	11.81	2.98	7.56	12.93	3.25	13.18	23.02	5.65
	SPONGY BONE	0.61	0.68	0.68	0.65	0.73	0.74	2.52	2.81	2.89
Triangular	CROWN	4.98	3.30	6.84	5.41	3.60	7.39	8.12	5.78	12.02
	ABUTMENT	27.79	8.55	60.12	28.04	8.63	60.64	53.42	15.90	119.62
	SCREW	10.17	16.83	16.72	10.73	17.87	17.97	16.73	29.08	30.50
	IMPLANT	36.69	33.07	17.47	39.82	35.80	19.04	71.75	61.40	34.79
	CORTICAL BONE	8.23	12.23	3.26	8.77	13.09	3.57	27.32	46.43	14.44
	SPONGY BONE	0.79	0.92	0.55	0.86	1.00	0.61	2.81	3.58	2.75

**Table 7:** Shows an ANOVA single factor statistical analysis for testing static and quasi-static loading difference on maximum stress in all implant thread shape.

ANOVA: Single Factor						
Summary						
Groups	Count	Sum	Average	Variance		
Static	30	3114.384	103.8128	9282.257		
Quasi-Static	30	3176.172	105.8724	9530.255		
ANOVA						
Source of Variation	SS	df	MS	F	*p*-value	F_crit_
Between Groups	63.62828	1	63.62828	0.006764	0.934734	4.006873
Within Groups	545,562.8	58	9406.256			
Total	545,626.5	59				

**Table 8:** ANOVA single factor statistical analysis for the effect of thread shape on maximum shear stress for the cyclic applied load.

Summary of One Way ANOVA						
Groups	Count	Sum	Average	Variance		
Square	6	111.93	18.655	314.408		
	6	85.67	14.27833	88.64706		
	6	107.71	17.95167	290.9025		
Buttress (CF)	6	119.8	19.96667	405.2682		
	6	86.35	14.39167	126.7028		
	6	151.19	25.19833	799.038		
Reverse Buttress (CF)	6	207.21	34.535	1021.174		
	6	185.83	30.97167	816.2047		
	6	209.49	34.915	888.1732		
Trapezoidal	6	162.69	27.115	862.3519		
	6	112.12	18.68667	260.4293		
	6	178.76	29.79333	894.9547		
Triangular	6	180.15	30.025	738.5722		
	6	162.17	27.02833	537.4403		
	6	214.12	35.68667	1833.773		
ANOVA						
Source of Variation	SS	df	MS	F	*p*-value	F_crit_
Between Groups	4664.334	14	333.1667	0.50592	0.922674	1.825908
Within Groups	49,390.2	75	658.536			
Total	54,054.53	89				
